# Two dominant patterns of low anterior resection syndrome and their effects on patients’ quality of life

**DOI:** 10.1038/s41598-021-82149-9

**Published:** 2021-02-11

**Authors:** Min Jung Kim, Ji Won Park, Mi Ae Lee, Han-Ki Lim, Yoon-Hye Kwon, Seung-Bum Ryoo, Kyu Joo Park, Seung-Yong Jeong

**Affiliations:** 1 Department of Surgery, Seoul National University College of Medicine, 101, Daehak-ro Jongno-gu, Seoul, 03080 Republic of Korea; 2 Cancer Research Institute, Seoul National University, Seoul, Korea

**Keywords:** Signs and symptoms, Gastroenterology, Gastrointestinal diseases

## Abstract

To identify low anterior resection syndrome (LARS) patterns and their associations with risk factors and quality of life (QOL). This cross-sectional study analyzed patients who underwent restorative anterior resection for left-sided colorectal cancer at Seoul National University Hospital, Seoul, Republic of Korea. We administered LARS questionnaires to assess bowel dysfunction and quality of life between April 2017 and November 2019. LARS patterns were classified based on factor analyses. Variable effects on LARS patterns were estimated using logistic regression analysis. The risk factors and quality of life associated with dominant LARS patterns were analyzed. Data of 283 patients with a median follow-up duration of 24 months were analyzed. Major LARS was observed in 123 (43.3%) patients. Radiotherapy (odds ratio [OR]: 2.851, 95% confidence interval [95% CI]: 2.504–43.958, p = 0.002), low anastomosis (OR: 10.492, 95% CI: 2.504–43.958, p = 0.001), and complications (OR: 2.163, 95% CI: 1.100–4.255, p = 0.025) were independently associated with major LARS. LARS was classified into incontinence- or frequency-dominant types. Risk factors for incontinence-dominant LARS were radiotherapy and complications, whereas those for frequency-dominant LARS included low tumor location. Patients with incontinence-dominant patterns showed lower emotional function, whereas those with frequency-dominant patterns showed lower global health QOL, lower emotional, cognitive, and social functions, and higher incidence of pain and diarrhea. Frequency-dominant LARS had a greater negative effect on QOL than incontinence-dominant LARS. These patterns could be used for preoperative prediction and postoperative treatment of LARS.

## Introduction

Rectal cancer survival rates have markedly improved as a result of advances in surveillance, surgery, and chemoradiotherapy. However, the functional consequences of treatment and quality of life (QOL) in survivors are often overlooked since post-treatment surveillance has mainly focused on recurrence. The use of stapling devices and neoadjuvant chemoradiotherapy has resulted in up to 80% of patients with rectal cancer to undergo sphincter-preserving surgeries (SPSs), and approximately 90% of these patients experienced bowel dysfunction following SPS^1^.

Common symptoms of bowel dysfunction following SPS include urgency, clustering, evacuation difficulty or incomplete emptying, and flatus or fecal incontinence. Over 40% of patients have been reported to become “toilet dependent,” which can have devastating consequences on a patients’ physical, social, occupational, and psychological functioning and significantly decreases their QOL^2,3^. The effects of bowel dysfunction that occur following surgeries for rectal cancer can persist even after long-term follow-up^4^.

Low anterior resection (LAR) syndrome (LARS) specifically refers to the bowel dysfunction following rectal cancer resection^5^. The evaluation of LARS varies considerably between studies, and the majority of previous studies focused on incontinence, rather than on other symptoms, such as frequency, clustering, incomplete emptying, and QOL. To overcome these limitations, the following two questionnaires were specifically developed to assess LARS symptoms: Memorial Sloan Kettering Cancer Center Bowel Function Instrument (MSKCC BFI) and LARS score^6,7^. The LARS questionnaire is shorter than the MSKCC BFI, and provides higher clinical utility by allowing rapid stratification of patients into no, minor, and major LARS groups. The LARS questionnaire was published in 2012, and translated versions have been validated internationally^8,9^.

Identifying patients at a high risk of major LARS is crucial when deciding between abdominoperineal resection (APR) with permanent colostomy and SPS procedures. Furthermore, classifying major patterns associated with LARS would be helpful in symptom management. However, even rectal cancer specialists may not be fully aware of LARS symptoms that patients consider to be the most bothersome; thus, they have difficulty identifying patients at risk for major LARS^10^. This study aimed to evaluate LARS risk factors using data gathered from patients undergoing long-term follow-up after rectal cancer surgery, and to identify patterns associated with LARS based on the major symptoms and their subsequent effects on QOL.

## Materials and methods

### Study design

This cross-sectional study was performed at the Seoul National University Hospital, Seoul, Republic of Korea and approved by the Institutional Review Board of this institution (IRB protocol number: 2007-012-1138). All research was performed in accordance with relevant guidelines/regulations. The requirement for obtaining patients’ informed consent was waived by the Institutional Review Board of this institution because the risk of this study on patients was minimal, and the information was collected for clinical use.

### Patients

Between April 2017 and November 2019, the LARS questionnaire was administered to patients in an outpatient clinic who underwent surgeries for restorative anterior resection (AR) for left-sided colorectal cancer. The inclusion criteria were those who underwent AR, LAR, and intersphincteric resections via open, laparoscopic, and robotic approaches. Patients who underwent trans-anal excision and total or subtotal colectomy were excluded.

### LARS questionnaire

Bowel function impairment was assessed using the LARS questionnaire, which assesses the following five items: incontinence for flatus, incontinence for liquid stools, frequency, clustering, and urgency. Each item carries three options with predefined scores used for evaluating severity. According to the total scores, patients were classified into the no (0–20), minor (21–29), and major (30–42) LARS groups. We used the validated Korean version of the questionnaire^11^. Correlations between the fecal incontinence severity index (FISI) and LARS groups were analyzed to validate the utility of the LARS questionnaire score.

Patient demographic, perioperative, and pathological data were obtained from the prospectively maintained database and compared among the no, minor, and major LARS groups. Variables demonstrating significant differences among the groups were used in the multivariable analysis to identify independent risk factors associated with major LARS.

### QOL questionnaires

The Korean versions of the European Organization for Research and Treatment of Cancer Quality of Life Questionnaire-C30 (EORTC QLQ-C30), European Organization for Research and Treatment of Cancer Questionnaire Module for Colorectal Cancer (EORTC QLQ-CR29), FISI, and Fecal Incontinence Quality of Life (FIQL) were used to evaluate the effects of LARS symptoms on postoperative QOL, and findings were compared among the three LARS groups.

### LARS pattern assessment

Patterns associated with LARS were determined using an exploratory approach with principal axis factoring analyses that were performed on the five LARS questionnaire items in the minor and major LARS groups. Extraction of the principal components was followed by varimax rotation to achieve a structure with independent factors and greater potential for interpretability. We used the minimum eigen values of 1.0, screen plot, and interpretability of the factors to determine which factors should be retained with regard to the LARS patterns. A factor score was calculated for each LARS pattern, and the score of each pattern was categorized into two quantiles, designated as low or high patterns. To identify the risk factors of minor and major LARS based on the observed patterns, the multivariable analysis was adjusted for potential risk factors found in the highest quintile of each LARS pattern.

### Statistical analysis

Continuous variables are presented as mean with standard deviations or median (minimum–maximum range) based on the normality of distributions, whereas categorical variables are presented as frequency (percentage). Continuous variables were compared using one-way analysis of variance or Kruskal–Wallis test, and categorical variables were compared using the chi-squared or Fisher’s exact tests.

Univariable and multivariable analyses were used to investigate the effects of each factor on major LARS, compared with no or minor LARS. The risk factors were estimated using odds ratios (ORs) calculated with logistic regression analyses. A backward selection model was used to select the variables for the multivariable model. All results with two-tailed *p-*values < 0.05 were considered to be statistically significant. The statistical analyses were performed using SPSS version 25 (IBM Inc., Armonk, NY, USA).

## Results

### Baseline demographics and tumor characteristics

Altogether, 283 patients were analyzed. There were 101 (35.7%), 60 (21.2%), and 123 (43.5%) patients in the no, minor, and major LARS groups, respectively, with median LARS scores of 13 (0–20), 26 (21–29), and 37 (30–41), respectively. The overall median time between surgery and questionnaire administration was 24 (range: 0–181) months. The stratified median time to questionnaire administration was 23 (range: 0–181), 20 (range: 0–120), and 28 (range: 0–115) months in the no, minor, and major LARS groups, respectively (p = 0.855).

Baseline demographic data were not significantly different among the three groups (Table 1). The tumors were located lower in the major LARS group than in the no and minor LARS groups (Table 2). Preoperative radiotherapy was administered more frequently in the major LARS group than in the other groups [14 (17.3%) no LARS, 13 (16.0%) minor LARS, and 54 (66.7%) major LARS group; p < 0.0001]. Major LARS was observed in 10 (15.2%), 82 (48.5%), and 31 (63.3%) patients who underwent AR, LAR, and ultralow anterior resection (ULAR), respectively (p < 0.0001). Diverting stoma formation was more frequently performed in the major LARS group [24 (24.2%) no LARS, 14 (14.1%) minor LARS, 61 (61.6%) major LARS; p < 0.0001). The anastomosis technique was not related to LARS occurrence (p = 0.165). The major LARS group had more postoperative complications than the other groups (p = 0.021). The mean time from surgery to stoma repair was not significantly different among the groups.

**Table 1:** Baseline characteristics.

	No LARS (n = 101)	Minor LARS (n = 60)	Major LARS (n = 123)	*p*
**Age, years (%)**				
Mean (SD)	61.7 (9.0)	58.4 (10.2)	58.9 (11.0)	0.067
≤ 60	51 (33.8)	32 (21.2)	68 (45.0)	0.774
> 60	50 (37.6)	28 (21.1)	55 (41.4)	
**Sex (%)**				0.961
Male	72 (35.8)	43 (21.4)	86 (42.8)	
Female	29 (34.9)	17 (20.5)	37 (44.6)	
**Mean BMI, kg/m**^**2**^ **(SD)**	23.9 (2.9)	23.9 (3.3)	23.9 (3.2)	0.987
**ASA class (%)**				0.918
1	33 (35.1)	21 (22.3)	40 (42.6)	
2	64 (35.6)	38 (21.1)	78 (43.3)	
3	4 (44.4)	1 (11.1)	4 (44.4)	
4	0 (0)	0 (0)	1 (100)	
**Diabetes (%)**				0.778
No	82 (34.9)	49 (20.9)	104 (44.3)	
Yes	19 (38.8)	11 (22.4)	19 (38.8)	
**Hypertension (%)**				0.318
No	60 (32.6)	39 (21.2)	85 (46.20	
Yes	41 (41.0)	21 (21.0)	38 (38.0)	
**Heart disease (%)**				0.716
No	95 (35.4)	57 (21.3)	116 (43.3)	
**Ischemic heart disease**	3 (30.0)	3 (30.0)	4 (40.0)	
Arrhythmia	3 (60.0)	0 (0)	2 (40.0)	
Both	0 (0)	0 (0)	1 (100.0)	
**Pulmonary disease (%)**				0.837
No	94 (35.9)	57 (21.8)	111 (42.4)	
Obstructive lung disease	1 (33.3)	1 (33.3)	1 (33.3)	
Tuberculosis	6 (35.3)	2 (11.8)	9 (52.9)	
Others	0 (0)	0 (0)	1 (100.0)	
**Liver disease (%)**				0.933
No	97 (35.3)	59 (21.5)	119 (43.3)	
Liver cirrhosis	3 (42.9)	1 (14.3)	3 (42.9)	
HBV hepatitis	1 (50.0)	0 (0)	1 (50.0)	
**Smoking (%)**				0.131
No	74 (36.8)	38 (18.9)	89 (44.3)	
Ex-smoker	11 (47.8)	7 (30.4)	5 (21.7)	
Current smoker	16 (26.7)	15 (25.0)	29 (48.3)	
**Alcohol (%)**				0.899
No	57 (35.0)	34 (20.9)	72 (44.2)	
Ex drinker	5 (50.0)	2 (20.0)	3 (30.0)	
Current drinker	39 (35.1)	24 (21.6)	48 (43.2)	

*LARS* low anterior resection syndrome, *SD* standard deviation, *BMI* body mass index, *ASA* American Society of Anesthesiology, *HBV* hepatitis B virus.

**Table 2:** Tumor characteristics and perioperative data.

	No LARS (n = 101)	Minor LARS (n = 60)	Major LARS (n = 123)	*p*
**Median pretreatment CEA (range)**	1.9 (0.4–185.6)	2.0 (0.4–181.0)	1.8 (0.4–125.4)	0.549
**Location of tumor (%)**				< 0.001
Sigmoid colon	37 (59.7)	13 (21.0)	12 (19.4)	
Rectosigmoid colon	15 (34.1)	15 (34.1)	14 (31.8)	
Rectum	49 (27.5)	32 (18.0)	97 (54.5)	
**Tumor location from the anal verge, cm (%)**^**a**^				< 0.001
15–20	36 (59.0)	15 (24.6)	10 (16.4)	
11–15	18 (31.0)	18 (31.0)	22 (37.9)	
6–10	33 (32.0)	15 (14.6)	55 (53.4)	
0–5	11 (19.6)	11 (19.6)	34 (60.7)	
**AJCC stage of tumor (%)**				0.206
0	1 (6.3)	3 (18.8)	12 (75.0)	
1	36 (37.9)	21 (22.1)	38 (40.0)	
2	27 (35.1)	17 (22.1)	33 (42.9)	
3	37 (38.5)	19 (19.8)	40 (41.7)	
**Tumor classification (%)**				0.169
T0	1 (8.3)	3 (25.0)	8 (66.7)	
Tis	0 (0)	0 (0)	5 (100.0)	
T1	25 (39.7)	13 (20.6)	25 (39.7)	
T2	18 (32.7)	10 (18.2)	27 (49.1)	
T3	50 (37.0)	31 (23.0)	54 (40.0)	
T4	7 (50.0)	3 (21.4)	4 (28.6)	
**Nodal classification (%)**				0.694
N0	64 (34.0)	41 (21.8)	83 (44.1)	
N1	32 (41.6)	14 (18.2)	31 (40.3)	
N2	5 (26.3)	5 (26.3)	9 (47.4)	
**Preoperative radiotherapy (%)**	14 (17.3)	13 (16.0)	54 (66.7)	< 0.001
**Operative name (%)**				< 0.001
Anterior resection	42 (63.6)	14 (21.2)	10 (15.2)	
Low anterior resection	48 (28.4)	39 (23.1)	82 (48.5)	
Ultralow anterior resection	11 (22.4)	7 (14.3)	31 (63.3)	
**Combined operation (%)**	31 (30.7)	21 (35.0)	60 (48.8)	0.016
No	70 (40.7)	39 (22.7)	63 (36.6)	
Pelvic organ resection/lateral lymph node dissection	5 (17.9)	8 (28.6)	15 (53.6)	
Others	26 (31.0)	13 (15.5)	45 (53.6)	
**Operative approach (%)**				0.147
Open	31 (30.4)	19 (18.6)	52 (51.0)	
Minimally invasive surgery	70 (38.5)	41 (22.5)	71 (39.0)	
Median operative time, min (range)	155.0 (63.0–390.0)	164.5 (60.0–396.0)	175.0 (56.0–528.0)	0.063
**Diversion (%)**	24 (24.2)	14 (14.1)	61 (61.6)	< 0.001
**Anastomosis (%)**				0.165
End-to-end double stapling	81 (38.4)	44 (20.9)	86 (40.8)	
Side-to-end double stapling	7 (21.9)	5 (15.6)	20 (62.5)	
End-to-end hand sewn	13 (31.7)	11 (26.8)	17 (41.5)	
**Mean postoperative hospital stay, days (SD)**	7 (4–30)	7 (4–14)	7 (4–30)	0.224
**Postoperative complication (%)**	13 (25.5)	7 (13.7)	31 (60.8)	0.021
Wound problem	3 (23.1)	1 (7.7)	9 (69.2)	0.144
Ileus	6 (35.3)	2 (11.8)	9 (52.9)	0.566
Deep organ infection	0 (0)	1 (20.0)	4 (80.0)	0.183
Anastomosis leak	0 (0)	0 (0)	4 (100.0)	0.070
Pulmonary problem	0 (0)	2 (25.0)	6 (75.0)	0.087
Urinary problem	1 (11.1)	2 (22.2)	6 (66.7)	0.254
Chyle ascites	1 (33.3)	1 (33.3)	1 (33.3)	0.866
Thrombocytosis	1 (100.0)	0 (0)	0 (0)	0.403
Stoma-related problem	1 (100.0)	0 (0)	0 (0)	0.403
Nerve injury	0 (0)	0 (0)	1 (100.0)	0.403
Renal problem	0 (0)	0 (0)	1 (100.0)	0.403
**Mean time from surgery to stoma repair, days (SD)**	182.3 (70.1)	170.1 (59.4)	210.3 (83.3)	0.119

*LARS* low anterior resection syndrome, *CEA* carcinoembryonic antigen, *AJCC* American Joint Committee on Cancer, *SD* standard deviation.

^a^278 patients.

### LARS questionnaire

**Figure 1:**
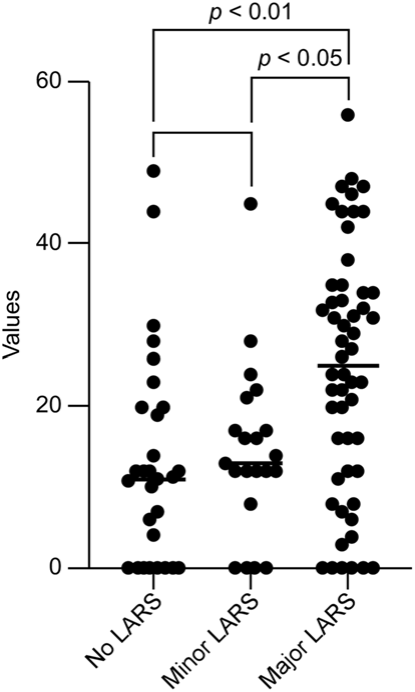
Fecal incontinence severity index according to the severity of low anterior resection syndrome. LARS, low anterior resection syndrome. Figure was generated using GraphPad Prism version 8.4.2 for macOS, GraphPad Software, San Diego, California USA, https://www.graphpad.com.

Except for the frequency subscale (p = 0.498), all other subscales were significant different among the three LARS groups (all p < 0.0167; see Table, Supplemental Digital Content 1, which displays all five subscales of the LARS questionnaire correlated with no, minor, and major LARS). The major LARS group had significantly higher FISI scores than the other groups (p = 0.0014 for no versus major LARS; and p = 0.0398 for minor versus major LARS, Fig. 1).

### Risk factors for LARS

In the multivariable analysis, preoperative radiotherapy (OR: 2.660, 95% confidence interval [CI]: 1.404–5.040, p = 0.003), LAR with low anastomosis (OR: 5.293, 95% CI: 1.825–15.348, p = 0.002), and postoperative complications (OR: 2.156, 95% CI: 1.103–4.214, p = 0.025) were independently associated with major LARS (Table 3).

**Table 3:** Multivariable analysis for risk factors of major low anterior resection syndrome.

Variables	OR	95% CI	*p*
**Preoperative radiotherapy**			
No	1		
Yes	2.660	1.404–5.040	0.003
**Operative name**			
Anterior resection	1		
Low anterior resection	4.511	1.972–10.323	< 0.001
Ultralow anterior resection	5.293	1.825–15.348	0.002
**Complication**			
No	1		
Yes	2.156	1.103–4.214	0.025

*OR* odds ratio, *CI* confidence interval.

### QOL and LARS

**Figure 2:**
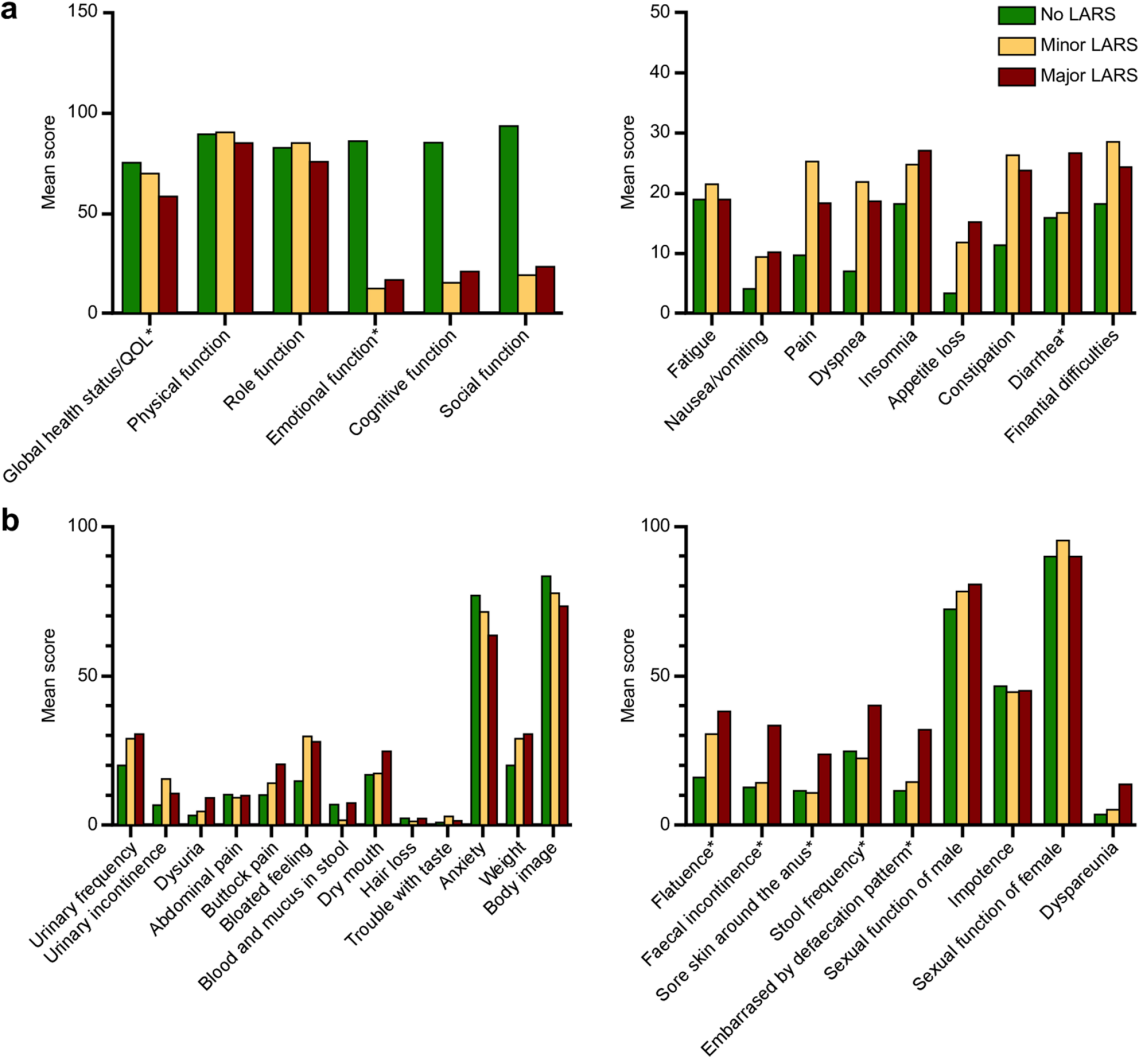
Quality of life according to no, minor, and major low anterior resection syndrome. **(a)** EORTC QLQ-C30 according to no, minor, and major low anterior resection syndrome. **(b)** EORTC QLQ-CR29 according to no, minor, and major low anterior resection syndrome. Asterisk: Overall p < 0.05. EORTC QLQ-C30, European Organization for Research and Treatment of Cancer Quality of Life Questionnaire; EORTC QLQ-CR29, European Organization for Research and Treatment of Cancer Questionnaire Module for Colorectal Cancer; LARS, low anterior resection syndrome; QOL, quality of life.

In the QOL analysis of 105 patients, the LARS groups with higher scores showed a lower global health status or QOL (p = 0.002), lower emotional function (p = 0.023), and a higher incidence of diarrhea (p = 0.028) in the EORTC QLQ-C30 than groups with lower scores (Fig. 2a). In the EORTC QLQ-CR29, defecation-related symptoms, including flatulence (p = 0.001), fecal incontinence (p < 0.001), sore skin around the anus (p = 0.015), stool frequency (p < 0.001), and embarrassed by defecation pattern (p < 0.001), were significantly higher in the major LARS group than in the no and minor LARS groups (Fig. 2b).

**Figure 3:**
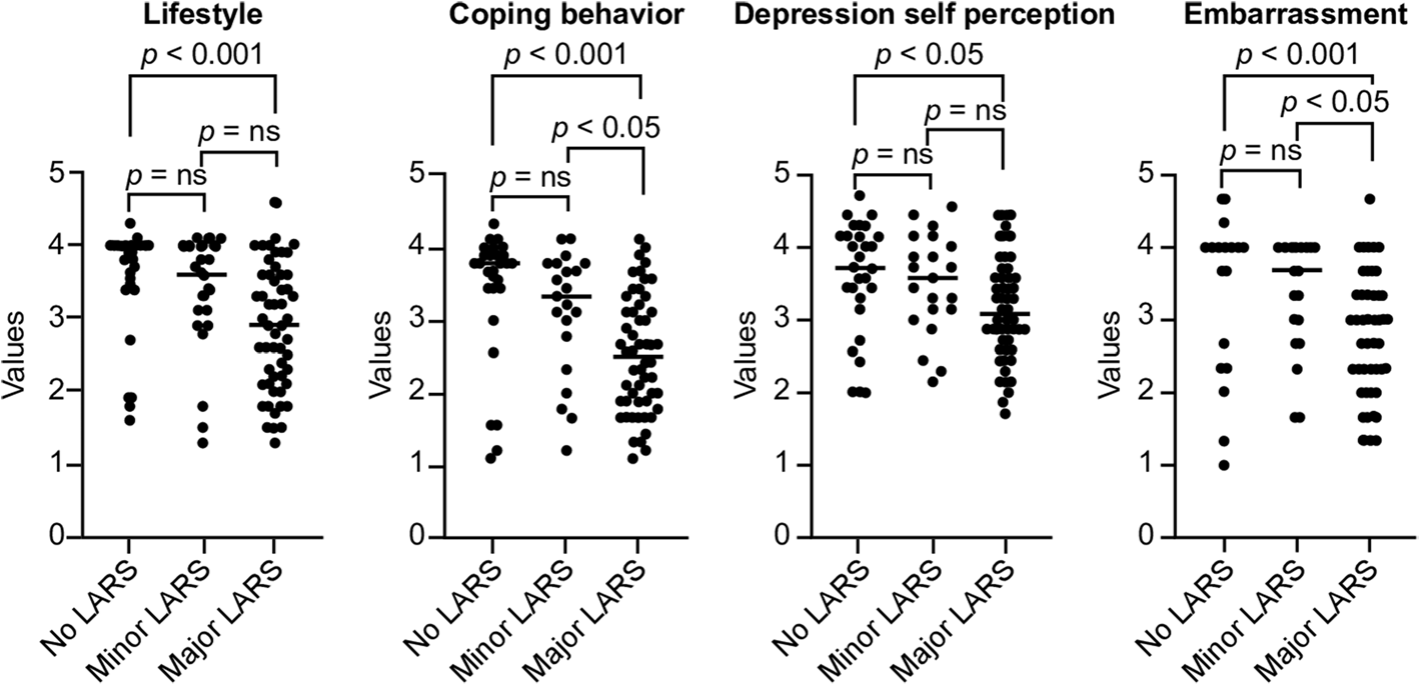
Fecal incontinence quality of life according to no, minor, and major low anterior resection syndrome. LARS, low anterior resection syndrome. Figure was generated using GraphPad Prism version 8.4.2 for macOS, GraphPad Software, San Diego, California USA, https://www.graphpad.com.

In the FIQL analysis, the major LARS group had worse QOL-related fecal incontinence in all four factors, including lifestyle, coping behavior, depression/self-perception, and embarrassment, compared to the no LARS group (all p < 0.05), and worse coping behavior (p = 0.0386) and embarrassment (p = 0.0221), compared to the minor LARS group (Fig. 3). No statistically significant differences in the four factors were observed between the no and minor LARS groups.

### LARS patterns

In patients with minor or major LARS, two dominant patterns were derived from the exploratory factor analysis and factor loading matrix using a rotated component matrix (See table, Supplemental Digital Content 2, which includes the factor loading matrix using a rotated component matrix for the two dominant patterns identified by factor analysis). The factors were interpreted as LARS patterns and named based on the LARS groups with high factor loading (Supplemental Digital Content 3). Pattern 1 was termed the incontinence-dominant pattern since it demonstrated high loading of incontinence LARS subscales, including incontinence for flatus and liquid stools. Pattern 2 was termed the frequency-dominant pattern because it demonstrated high loading for symptoms related to frequency, including subscales of frequency, urgency, and clustering.

### Risk factors associated with LARS patterns

After dividing the patients into the low- or high-score groups based on each LARS pattern, ORs (95% CI) were obtained to explain the risk factors associated with each LARS pattern after adjusting for potential cofounders (Table 4). The adjusted variables entered in the analysis were age, sex, ASA class, tumor location from the anal verge, type of surgery, combined surgeries, diversion, postoperative complications, AJCC tumor stage, and surgical approach. Preoperative radiotherapy and postoperative complications were associated with incontinence-dominant LARS, whereas tumor location from the anal verge were associated with frequency-dominant LARS.

**Table 4:** Multivariable analysis for risk factors of minor or major LARS according to LARS patterns.

Variables	OR	95% CI	*p*
***Pattern 1: incontinence dominant LARS***			
**Preoperative radiotherapy**			
No	1		
Yes	3.334	1.745–6.369	< 0.0001
**Complication**			
No	1		
Yes	2.194	1.012–4.757	0.047
***Pattern 2: frequency dominant LARS***			
**Tumor location from the anal verge, cm (%)**			
15–20	1		
11–15	2.528	0.719–8.888	0.148
6–10	9.450	2.916–30.622	< 0.0001
0–5	8.647	2.534–29.506	0.001

*LARS* low anterior resection syndrome, *CI* confidence interval, *OR* odds ratio.

### QOL and LARS patterns

**Figure 4:**
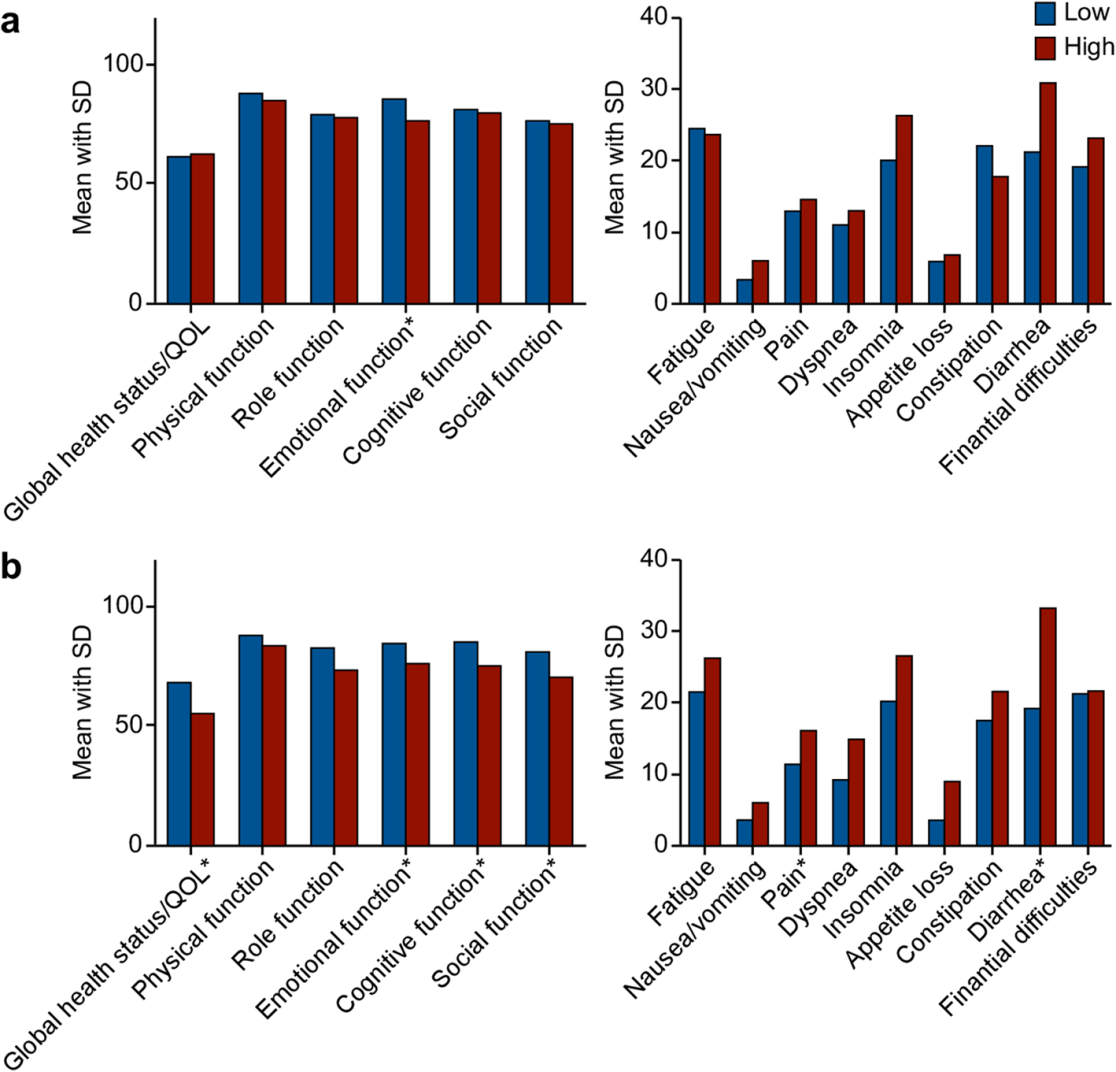
EORTC QLQ-C30 according to low anterior resection syndrome patterns. **(a)** EORTC QLQ-C30 according to low and high incontinence-dominant low anterior resection syndrome. **(b)** EORTC QLQ-C30 according to low and high frequency dominant low anterior resection syndrome. Asterisk: Overall p < 0.05. EORTC QLQ-C30, European Organization for Research and Treatment of Cancer Quality of Life Questionnaire; EORTC QLQ-CR29, European Organization for Research and Treatment of Cancer Questionnaire Module for Colorectal Cancer; SD, standard deviation.

To analyze the effects of each LARS pattern on a patient’s QOL, the EORTC QLQ-C30 scores were compared between the low- and high-score groups (n = 76, Fig. 4). Only emotional function was associated with the incontinence-dominant LARS pattern, whereas global health status/QOL, emotional and social functions, pain, and diarrhea were associated with the frequency-dominant LARS pattern.

## Discussion

Our study found that preoperative radiotherapy, low rectal cancer, and postoperative complications were independent risk factors for major LARS. LARS was classified into incontinence- and frequency-dominant patterns, with each pattern being related to different risk factors. The incontinence-dominant pattern was related to preoperative radiotherapy and postoperative complications, whereas the frequency-dominant pattern was related to a low tumor location from the anal verge. Overall, major LARS was associated with a worse QOL, and frequency-dominant LARS had more profound effects on postoperative QOL.

LARS attenuates the relative SPS benefits achieved by preserving the anus when compared to APR. SPS restores bowel continuity, yet there is no clear evidence available with regard to the differences in QOL between patients who underwent SPS and those who underwent APR ^3,12,13^. The similarities in patient-reported QOL may be partly attributed to postoperative bowel dysfunction, since 50%–90% of patients who undergo SPS for low rectal cancers with anastomosis close to the anus experience some degrees of postoperative bowel dysfunction. Preoperative radiotherapy is frequently performed in patients with mid-to-low rectal cancers, and postoperative complications, such as anastomosis leakage, are more common in low-lying tumors following radiotherapy, which exacerbates postoperative bowel dysfunction.

In general, LARS improves over time, especially within 6 months postoperatively. However, afterwards, LARS symptoms and the impact on patients’ QOL persist over time ^4^. Pieniowski et al. measured LARS and QOL questionnaires at 2 time points and found that there was no significant difference. In Denmark study, the time since surgery showed no association with major LARS (OR = 0.78, 95CI = 0.59–1.04)^14^. A review on LARS commented that LARS results in permanent changes rather than short-lived neorectal irritability in the postoperative period^1^. Although we did not measure LARS score twice in the same patients, the prevalence of major LARS score were not significantly different over time.

Low anastomosis and preoperative radiotherapy are the two main factors for postoperative bowel dysfunction and have been previously reported as independent risk factors^6,13–17^. In a cross-sectional study by Trenti et al., neoadjuvant radiotherapy (OR: 2.38, p = 0.048) and coloanal anastomosis (versus colorectal anastomosis, OR: 3.82, p = 0.005) were identified as significant risk factors for major LARS^13^. Sun et al. reported that long-term preoperative radiotherapy (OR: 2.20, p = 0.007) and anastomosis height (OR: 0.74, p < 0.001) were also independent risk factors for major LARS^18^. A cross-sectional multi-center cohort study identified major LARS in 60% of patients with low rectal cancer treated with preoperative radiotherapy compared to only 33% of patients with middle or upper rectal cancers without preoperative radiotherapy^15^. In a meta-analysis that analyzed 11 studies between 2005 and 2017, radiotherapy in either preoperative or postoperative regimens were the most significant risk factor for major LARS in eight studies, and tumor height (anastomosis level) was the second most significant risk factor of major LARS in six studies^16^.

Identification of LARS risk factors allows for the prediction of the degree of postoperative bowel dysfunction and QOL impairment, which can help preoperatively counsel patients regarding the decision of preserving the sphincter. In our study, 65.9% and 46.7% of patients with rectal cancer located within 5 cm of the anal verge with and without preoperative radiotherapy, respectively, developed major LARS, whereas 26.7% and 16.4% of patients with rectal cancer located > 10 cm from the anal verge with and without preoperative radiotherapy, respectively, developed major LARS. The pre-operative LARS score (POLARS) nomogram was developed by the United Kingdom and Danish LARS study group to predict the risk for LARS occurrence. Predictive factors used in the POLARS are age, tumor height, total mesorectal excision versus partial mesorectal excision, stoma, and preoperative radiotherapy, with tumor height and preoperative radiotherapy having the greatest contribution to a high expected LARS score^19^. Only preoperative factors were used in POLARS, because it was developed for efficient preoperative prediction of LARS; thus, the postoperative complications that were found to be significant risk factors for LARS in our study were not considered in the design of POLARS. A stoma was believed to be related to disuse colitis, which might result in bowel dysfunction following stoma repair^20^; however, stoma was not a significant risk factor in our multivariable analysis, and the time interval from SPS and stoma repair was not related to a higher prevalence of major LARS. Stoma may be a confounding factor with low anastomosis and preoperative radiotherapy, and stoma-related bowel dysfunction may be transient following stoma repair.

In the original study that developed and validated LARS questionnaire, there was a significant difference in QOL between no, minor, and major LARS group^7^. But, in this study, a simple question, “On overall, how much is your QoL influenced by your bowel dysfunction?”, was used to assess the impact of LARS on QOL. In a subsequent study involving 5 centers in 4 European countries, EORTC QLQ-C30 was used, and from analysis of 796 patients, patients with major LARS showed worse QOL in all subscales except constipation^2^. Although a few scales showed significant differences between no and minor LARS, the differences were too small to be considered clinically relevant, and therefore, the authors commented that no and minor LARS groups could be regarded comparable, and major LARS are the ones who need attention. These findings were in line with our results.

The challenges of LARS treatment may be due to the poor understanding of the underlying mechanisms and consequent variety of associated symptoms. In 2019, McKenna suggested that the treatment choice for LARS should be based on the predominant symptoms, and minor LARS cases were classified into diarrhea-dominant, incontinence-dominant, and urgency-dominant groups^21^. In this treatment algorithm, diarrhea-predominant minor LARS can be treated with anti-diarrheal and bulking agents, and incontinence- and urgency-predominant minor LARS can be treated with serotonin-3 receptor antagonists. Major LARS was not classified in that study, and suggested treatments included transanal irrigation, sacral nerve stimulation, and transition to a permanent stoma. However, this classification was not based on patient data and mainly focused on currently available treatment options, rather than on symptoms with major effects on QOL. A previous review article provided two pragmatic definitions of LARS symptoms, including urgency or fecal incontinence and evacuatory dysfunction^1^. It suggested that urgency or fecal incontinence might be related to anal sphincter damage and preoperative radiotherapy; both symptoms corresponded to our incontinence-dominant LARS. The other main symptom, evacuatory dysfunction, is a possible consequence of impairments in the autonomic nerve supply to the rectum, resulting in a loss of rectal coordination and paradoxical anal contraction. Patients with LARS often have frequent bowel movements because of tenesmus caused by incomplete emptying, and these symptoms also relate to clustering, defined as the bowel evacuation within an hour of the previous evacuation, leading to the complaint of frequent bowel movements. Therefore, the second classification using evacuatory dysfunction corresponds to our frequency-dominant LARS classification. Although evacuatory dysfunction has not been as well studied as the previously mentioned incontinence pattern, the symptoms of frequency and clustering had more negative effects on patient QOL than symptoms of incontinence symptoms in our study. Therefore, future studies should focus on these symptoms and associated treatments.

Our study has several strengths. It is the first study to classify the minor and major LARS types based on patient data. It also demonstrates that the different patterns are related to varying risk factors with diverse effects on QOL. Follow-up studies are warranted to validate our findings in other datasets to allow for patient care to be focused on observed LARS patterns. Second, many risk factors that could be associated with major LARS were comprehensively analyzed. From our prospective database, we extracted as many variables as possible for analysis as possible risk factors.

This study also has several limitations, including the lack of baseline data available on patient LARS status and QOL measures. A recent report on normative LARS data found that even patients who did not undergo surgery could present with LARS symptoms^22^. Therefore, future studies should collect baseline data to better distinguish the effects of surgery on bowel dysfunction. Second, the medication information about antidiarrheal medications were not collected in this study. Antidiarrheal medications such as loperamide were frequently prescribed in LARS patients and could affect the frequency score in LARS questionnaire. Patients are often prescribed not only in our hospital, but also in other clinics, and take the drug by adjusting its dose according to their symptoms. Therefore, to collect accurate data on LARS medications, data should be collected prospectively or from nationwide prescription database.

In conclusion, we demonstrated that the risk factors associated with major LARS include preoperative radiotherapy, sphincter-saving surgery with low anastomosis, and postoperative complications. We classified LARS into either incontinence-dominant or frequency-dominant patterns based on symptoms. The incontinence-dominant pattern was related to preoperative radiotherapy and postoperative complications, whereas the frequency-dominant pattern was related to low tumor location. Frequency-dominant LARS had more profound associations with poor QOL. Ultimately, using these new LARS classifications could be useful in LARS management.

## Supplementary Information

Supplementary Information.
