# Supplementary material for: Two dominant patterns of low anterior resection syndrome and their effects on patients’ quality of life
Source: Sci Rep. 2021 Feb 11;11:3538. doi: 10.1038/s41598-021-82149-9 (PMC7878496; doi:10.1038/s41598-021-82149-9)
Supplement: Supplementary file 1 — Supplementary Information. [file 41598_2021_82149_MOESM1_ESM.docx]

**Two Dominant Patterns of Low Anterior Resection Syndrome and their Effects on Patients’ Quality of Life**

**Running Head:** Low anterior resection syndrome patterns

Min Jung Kim, MD^1,2^, Ji Won Park, MD, PhD^1,2^, Mi Ae Lee, MS^1^, Han-Ki Lim, MD^1^, Yoon-Hye Kwon, MD^1^, Seung-Bum Ryoo, MD, PhD^1^, Kyu Joo Park, MD, PhD^1^, and Seung-Yong Jeong, MD, PhD^1,2^

^1^Department of Surgery, Seoul National University College of Medicine, Seoul, Korea

^2^Cancer Research Institute, Seoul National University, Seoul, Korea

**Corresponding author:**

Ji Won Park, MD, PhD

Department of Surgery, Seoul National University College of Medicine

101, Daehak-ro Jongno-gu, Seoul 03080, Republic of Korea

Tel.: +82-2-2072-7211

Fax: +82-2-741-0548

E-mail: sowisdom@snu.ac.kr

Supplemental Digital Content 1. Low anterior resection syndrome according to questionnaire subscales

|  | No LARS (n=101) | Mild LARS (n=60) | Major LARS (n=123) | p |
| --- | --- | --- | --- | --- |
| 1. Incontinence for flatus (%) |  |  |  | <0.001 |
| No, never (0) | 66 (56.9) | 28 (24.1) | 22 (19.0) |  |
| Yes, less than once per week (4) | 20 (37.0) | 15 (27.8) | 19 (35.2) |  |
| Yes, at least once per week (7) | 15 (13.2) | 17 (14.9) | 82 (71.9) |  |
| 2. Incontinence for liquid stools (%) |  |  |  | <0.001 |
| No, never (0) | 86 (55.8) | 39 (25.3) | 29 (18.8) |  |
| Yes, less than once per week/Yes, more than once per week (3) | 15 (11.5) | 21 (16.2) | 94 (72.3) |  |
| 3. Frequency (%) |  |  |  | <0.001 |
| 1–3 times per day (0) | 64 (53.8) | 32 (26.9) | 23 (19.3) |  |
| 4–7 times per day (2) | 18 (22.5) | 19 (23.8) | 43 (53.8) |  |
| More than 7 times per day (4) | 3 (5.4) | 5 (8.9) | 48 (85.7) |  |
| Less than once per day (5) | 16 (55.2) | 4 (6.7) | 9 (31.0) |  |
| 4. Clustering (%) |  |  |  | <0.001 |
| No, never (0) | 35 (92.1) | 3 (7.9) | 0 (0) |  |
| Yes, less than once per week (9) | 31 (47.7) | 18 (27.70 | 16 (24.6) |  |
| Yes, at least once per week (11) | 35 (19.3) | 39 (21.5) | 107 (59.1) |  |
| 5. Urgency (%) |  |  |  | <0.001 |
| No, never (0) | 90 (90.9) | 9 (9.1) | 0 (0) |  |
| Yes, less than once per week (11) | 11 (14.5) | 39 (51.3) | 26 (34.2) |  |
| Yes, at least once per week (16) | 0 (0) | 12 (11.0) | 97 (89.0) |  |

Abbreviation: LARS=low anterior resection syndrome

Supplemental Digital Content 2. Factor loading matrix using a rotated component matrix for the two dominant patterns identified by factor analysis

|  | Factor loadings | |
| --- | --- | --- |
|  | Pattern 1 | Pattern 2 |
| Incontinence for flatus | 0.796 | –0.162 |
| Incontinence for liquid stools | 0.812 | 0.190 |
| Frequency | 0.004 | 0.760 |
| Clustering | 0.190 | 0.404 |
| Urgency | –0.154 | 0.624 |

Extraction method: Principal component analysis

Rotation method: Varimax with Kaiser normalization

Supplemental Digital Content 3. Component plot in rotated space displaying the items of the low anterior resection syndrome questionnaire.^§^


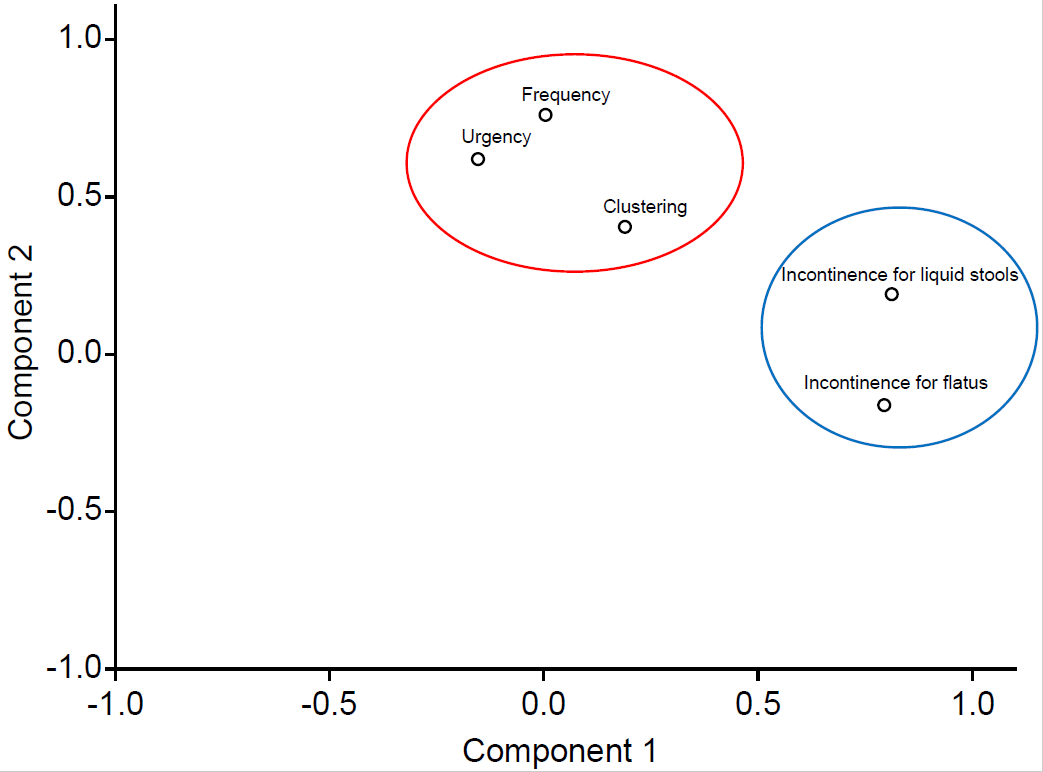


^§^Figure was generated using IBM SPSS Statistics for Windows, Version 25.0, Armonk, NY, IBM Corp., www.ibm.com
